# Health-Resorts in the West of England and South Wales

**Published:** 1891-12

**Authors:** Walter Tyrrell

**Affiliations:** Medical Officer of Health for Great Malvern


### Health-Resorts in the West of England and South Wales.

V.

### MALVERN.

BY

WALTER TYRRELL, M.R.C.S. Eng., L.S.A.,
*Medical Officer of Health for Great Malvern.*

THERE is a well-known Midland County proverb which boldly asserts that—

> "On Malvern Hill
> A man may live as long as he will."

Now, although unable to endorse this statement as absolute truth, yet I think I shall be able to show in the following pages that the Malvern range can claim advantages of position, soil, and atmospheric conditions which render it not merely of value as a health-resort, but also singularly efficacious in remedying certain constitutional defects, and restoring to tone and vigour organs whose defaults of function have been brought about from over-work, mental distress, or residence in low-lying or malarial districts.

I shall endeavour first to show in what particulars Malvern differs from almost all other health-resorts, and in what its peculiar advantages consist: this done, I shall seek to point out those diseases which I have found to be most benefited by the climate and general surroundings, and the manner in which such favourable results are obtained.

The Malvern range of hills extends for eight or nine miles in a direction nearly due north and south. The North Hill, which terminates the range in that direction, is rather over 1300 feet in height, and is surrounded by houses which line the road, which, gradually ascending, winds round it from east to west.

Great Malvern lies 500 feet above the sea on the eastern side of the range, in a somewhat sheltered valley between the North and Beacon Hills. At its back the Worcestershire Beacon rises to a height of 1400 feet.

Although it is principally of Great Malvern that I purpose treating, yet I may here mention that West Malvern, which is situate 850 feet above the sea, possesses great advantages at certain times of the year. In summer it is especially favoured by its elevated position and exposure to the soft west winds. Lying as it does close under the brow of the hill, it is thoroughly sheltered from the east and north. Its great altitude also keeps it free from fogs and mists.

Malvern Wells, lying to the south of Great Malvern, is thoroughly sheltered, and possesses much the same salubrious conditions as the latter town.

I may briefly note the favourable conditions which have combined to make the climate of Malvern so excellent for the treatment of many forms of disease. These are (1) its aspect, (2) its elevation, and (3) the nature of its soil.

From its situation high up on the eastern side of the Malvern range, and having no hill in front of it by which mists and moisture can be retained, it follows that all cloud and wet which accompany the west and southerly winds, striking the hill on the west side, are thrown upwards, and passing over the town, are blown completely away, thus ensuring a marked absence of moisture. Again, the elevation and easterly aspect of the town ensure the incidence of the earliest sun-rays, which, poured on the hillside, are retained during the day by the sparsely-covered Syenite which forms its bulk, to be radiated forth so soon as the chills of evening set in, thus ensuring the very slight variation between the day and night temperatures—a feature of the climate which is as advantageous as it is remarkable.

Again, as the town is built in a recess or valley on the hillside, not thrust forward on a spur or projection, it is naturally much sheltered, especially from the east wind; for a phenomenon similar to that which I have described as occurring on the west side of the hill, gives us an immunity from this "wind of God," as Kingsley somewhat inaptly terms it. In this case the easterly wind strikes the foot of the hill, and is thus thrown upward and passes over our heads. In the quarter of a century, during which I have resided in Great Malvern, I have never seen any damage which resulted from an easterly or north-easterly gale; while the southerly and south-westerly gales, from which we would appear to be quite protected, often thunder down our hillside, driven by the pressure of the gale as it tops the hill, and carry destruction to roof and tree.

Naturally, Malvern owes much to its elevation; to this, and partly also to the somewhat precipitous character of the rise, it owes its very complete and rapid drainage: even when rain has fallen for many consecutive hours, two or three hours' cessation, with a sun and breeze, will suffice to set the dust blowing.

So far as temperature is concerned, it is not difficult to explain why Malvern is so much warmer than many other places, apparently so much more favoured by situation: the equability and moderation of our temperature is, no doubt, mainly due to the absence of moisture from the air, and the supply of dry heat which is constantly furnished by that vast firebrick of Syenite at our back. Whatever the cause, the fact remains that if a thermometer be carried from the level of the town to the valley below, it will be found to fall from 8 to 10 degrees in the course of its transit; and Cheltenham, which is situated in a sheltered valley about twenty miles from Malvern, is always from 10 to 12 degrees colder than Great Malvern in winter. No doubt many of the important characteristics of Malvern air are due to the nature of its soil, which, consisting chiefly of rocky *débris* eroded from the hill, is porous, light, and dry; while the great mass of Syenite upon which it rests acts as a most effective filter, and supplies us with the purest and most delicious water in England.

The freedom from fog and mist, which is so happy a feature of the climate of Malvern, is certainly due to the favourable combination of aspect, elevation, and soil. In the cold weather which usually succeeds the rains of autumn, and when fogs and dense mists enfold most parts of England in gloom and darkness, Malvern is usually bright and sunny; indeed, November and December are among our best months, and we often rise with a bright blue, sunny sky above us, to find the valley below wrapped in a white sea of fog.

Of course the absence of moisture robs the cold of its rigours. It is a common remark of visitors that they never feel the cold in Malvern.

I will first recapitulate those peculiar advantages which the air of Malvern affords, and will then endeavour to point out the diseases to the cure or alleviation of which these conditions are advantageous.

(1) The dryness of the air and its highly-oxygenated and consequently invigorating properties; the dryness of the soil, so that out-door exercise is almost always possible.

(2) The large amount of sun and the immunity from mist and fog, so that the functions of respiration and consequently of vascular circulation are carried on with greater ease.

(3) And, perhaps, most important is the equable nature of the temperature, the variation between the night and day measurements being unusually small.

We now come to the main purpose of this paper; viz., to endeavour to show what forms of disease are most benefited by the atmospheric conditions which I have enumerated.

Congestion is a disorder which manifestly precedes and induces many forms of disease. More especially is this the case where the lungs and air-passages are concerned. In most cases, no doubt, this tendency to local congestion is due to natural deficiency or failure of the vigour and circulation of nerve force; so that not only does the vascular circulation suffer from a deficient *vis a tergo*, but there is also a marked default in the power of resistance to outside causes, such as cold and damp.

I would point out, then, that it is in disordered conditions of the vascular system that Malvern air is especially beneficial. One of the most common forms under which we recognise this default, is in the various stages of congestion which precede and accompany the deposit of tubercle, and it is precisely in these initiatory conditions that the greatest benefit is to be gained.

In phthisis—and I take this as the most fatal form of lung disease—there is, as in all other disorders, a natural tendency to recovery, and many cases do undoubtedly get well even under the most adverse circumstances, a fact which will be conceded by all who have had much experience in examinations of the body after death : the number of cases in which old-standing disease of the lungs is found to have undergone a process of natural cure is very large.

This fact should encourage us to endeavour to secure for those in whom such indications of disease exist the most favourable vital conditions. It is, of course, of primary importance that such patients should breathe an atmosphere not only free from mechanical and chemical impurities, but also of equable temperature, and as far as possible free from humidity: it is positive, from ascertained facts, that it is under such conditions that the greatest immunity from phthisis is found. It is, then, to climatic influence that we must first look in the treatment of lung disease; and it is to this that all other plans of treatment must be subservient. Medicine, for instance, can never be more than an adjuvant.

To stay the oncoming of disease is, of course, the first point ; but even when once established, much may be done to aid Nature in the reparative process which she is always endeavouring to set up. In both these alternatives, climate is of the greatest importance. The antecedent stages of phthisis are almost all of a congestive type, usually accompanied by more or less febrile action ; there is an accelerated pulse and heart action, and a rapidity of combustion which leads to rapid loss of fatty matter. Now, it is this early stage of the disorder that a dry cold air is so successful in combating: the continued respiration of a bracing tonic air leads to a contraction of the vascular system of the mucous membrane, and a consequent abatement of the congestion. Another quite as effective, though less apparent, reason for this result is, no doubt, the tonic action which dry cold has on the nervous circulation: under such conditions there naturally ensues a subsidence of the febrile action.

What I have said above respecting phthisis applies with equal force to all those states of congestion which, though more passive in their nature, attend upon and keep up chronic bronchitis. A most marked instance of the action of this climate on such a case came under my care two years ago. A gentleman of middle age had suffered for many years from continued attacks of bronchitis, with the utmost difficulty of breathing, and accompanied by more or less spasmodic asthma. There was, as the attack subsided, the most profuse muco-purulent expectoration, his vital power was low, and his emaciation extreme. In 1889 I attended him for the first time through a most severe attack. I advised a trial of a residence in Malvern; he having previously tried almost all climates, home as well as foreign. Since that date he has only had one seizure of any importance. He passed through the rigours of last winter with impunity. He has gained considerably in flesh, and really enjoys very good health.

Patients who come to Malvern suffering from the conditions which I have endeavoured to describe often make great gains in flesh. I often find such cases gain from two to six pounds in weight in the month. In two cases which have come here this autumn,—in the first of which there existed the antecedent symptoms of phthisis; in the second, the deposit of tubercle had actually taken place,—the first case gained over four pounds of flesh in the month; the latter, eight. Nor are such results at all uncommon. In both cases there was a corresponding improvement in the general health.

There are certain conditions which have their origin in defective nervous control, and which are to be met with in young persons of either sex, in which the benefits to be derived from a residence in Malvern air are so decided, that I make no apology for particularising them. The symptoms are those of general vital depression, the most evident signs of which are the derangement of the functions of most of the abdominal organs. These patients, who are often at or about the period of puberty, become full-bellied; the limbs lose their firm muscularity, and become wasted and flabby; the complexion becomes dull, and the skin damp and clammy. They are readily wearied, and complain of a corresponding mental fatigue; they are also irritable and peevish. In this state, or rather in the condition following upon it, the glands are liable to become enlarged and take on unhealthy inflammation. The liver is often enlarged and tender, and the bowels, in consequence, constipated and irregular. Now, these states are more readily benefited by a change to a dry, bracing air than by any other method.

The results of depraved nutrition are often manifested by sensation sometime before they become evident by any physical change. A sense of languor, and want of power and energy, is often complained of. After a time the patient loses flesh, and the features become pinched and faded; the eye loses its wonted expression, except under excitement or at night, when its increased brilliancy and enlarged pupil bespeak febrile excitement and nervous irritability. The mouth becomes drawn and the alæ nasi are dilated. With all these palpable changes, the functions of almost all internal organs become impeded; sluggish action of the bowels is common, alternating at times with irritable diarrhœa; the menstrual epoch is likely to be irregular, often delayed, at times altogether suspended; the urine deposits lithates, and many dyspeptic symptoms are apt to occur.

The febrile irritability is manifested by a pulse of from 80 to 100, increasing towards evening; at the same time these changes are often coincident with a marked deterioration in the quality of the blood, with a great deficiency of red particles.

The mental state coincides exactly with these physical conditions: the morning is all lassitude and languor; the evening, excitement and feverish restlessness. Insomnia is also often a prominent symptom.

This is a fairly true picture of the states which precede phthisis—states which are undoubtedly most surely relieved by change of climate. But, in the multitude of counsellors, which climate is most likely to cure the patient? Some recommend the high altitudes of Davos-Platz or St. Moritz; others, again, pin their faith on the Riviera, or those sheltered health-resorts which lie in the South of France or the valleys of the Pyrenees. Others, again, advise a voyage and a residence, longer or shorter, in South America or Australia. All these schemes have their advantages in certain cases; all have the possibility of still greater disadvantage. And it is rare to find two cases to which the same method, or a like change of climate, is applicable.

The heroic measure of transplanting your patient into the highly-rarefied air and extreme cold of the very high altitudes is applicable only to such cases as are in their earliest stages, and have still a large amount of vigour of constitution. The Riviera has its dangers in rapid changes of climate, sudden or extreme variations of temperature, and an almost complete absence of any sanitary rule; and, unless the patient be wealthy, to these must be added the entire change from a nourishing plain diet to bad meat, worse cooked. And, again, the tedious length of journey, the loss of home and its familiar surroundings, and a possible ignorance of the language spoken, should all be taken into account. "*Cœlum, non animum, mutant qui trans mare currunt;*" and the *mal de pays* is probably more strong in the disordered than in the healthy subject.

In conclusion, I cannot do better than quote the words of the late Dr. J. Henry Bennet, who, writing on this point, says: "For summer, Alpine elevations in Europe are, no doubt, an immense improvement on the heated plains and cities of Continental Europe, and it is easy to understand the rush made to them by the Germans and Swiss; but are they superior in climate and health advantages to the cool, breezy shores and hills of Great Britain for invalids? I doubt it."

The stimulating effect of cold upon the vascular and nervous systems needs no demonstration here; but the amount of vitality which may be conveyed into the system by the constant inspiration of a cold dry air, largely charged with oxygen, can hardly be imagined.

There is a well-known form of congestion of the pharynx and larynx which, from the frequency with which it occurs in members of the profession, has been termed "Clergyman's throat." It is ordinarily due to over-exertion of the vocal organs in vitiated atmospheres or damp relaxing climates: it is at times accompanied by a gouty state of the blood.

On examination, the mucous membrane of the back of the pharynx is found to be thickened, of a deep crimson colour, and traversed by enlarged and tortuous vessels. The laryngoscope shows more or less relaxation of the vocal cords. Now, to watch the curative effect of the dry cold air upon such cases is most gratifying. I have every winter several such cases under my care, and am always much interested in watching the certainty and rapidity with which a return to a healthy condition comes on.

Meteorological data, unless they extend over a very lengthened period of observation and embrace a wide observation of phenomena, must be more or less open to fallacy; but the evidence which Nature supplies in the forms of vegetable life with which she clothes more favoured spots, is of undeniable value.

In Malvern, the beauty and luxuriance of the growth of the evergreens, many of them of a most delicate nature, is, I believe, unsurpassed; thus affording incontestable evidence of the dry, mild, and equable climate. This positive evidence is, however, well borne out by the meteorological statistics, which afford evidence strongly confirmatory of the views I have here expressed. Space will not permit me to extend a paper which has already run to a rather unconscionable length.

It is out of the fulness of the heart that the mouth speaketh; and holding very strong views on the eligibility of the Malvern range as a health-resort for all patients who have in any way lost their nervous vigour, I have no hesitation in pressing upon the profession a recognition of its virtues.

